# Developing and Validating Novel Nomograms for Predicting the Overall Survival and Cancer-Specific Survival of Patients With Primary Vulvar Squamous Cell Cancer

**DOI:** 10.3389/fmed.2021.777605

**Published:** 2021-12-03

**Authors:** Weili Zhou, Yangyang Yue

**Affiliations:** ^1^Department of General Surgery, Shengjing Hospital, China Medical University, Shenyang, China; ^2^Department of Health Management, Shengjing Hospital, China Medical University, Shenyang, China

**Keywords:** invasion depth, lymphadenectomy, sentinel lymph node biopsy, nomogram, tumor size, vulvar cancer, N stage, M stage

## Abstract

**Background:** To develop and validate novel nomograms for better predicting the overall survival (OS) and cancer-specific survival (CSS) of patients with vulvar squamous cell cancer (VSCC).

**Methods:** A retrospective analysis using a population-based database between 2004 and 2016 was carried. A 10-fold cross-validation with 200 repetitions was used to choose the best fit multivariate Cox model based on the net-benefit of decision curve analysis. Net-benefit, Harrell's C concordance statistic (C-statistic) of calibration plot, and area under the receiver operating characteristic curve (AUC) were used to evaluate the model prediction accuracy. Nomograms of the OS and CSS were generated based on the best fit model.

**Results:** Of the 6,792 patients with VSCC, 5,094 (75%) and 1,698 (25%) were allocated to the training and validation cohort, respectively. All the variables were balanced between the training and validation cohorts. Age, insurance, tumor size, pathological grade, radiotherapy, chemotherapy, invasion depth, lymphadenectomy, sentinel lymph nodes biopsy, surgery, N stage, and M stage were in the best fit model for generating nomograms. The decision curve analysis, calibration plot, and receiver operating characteristic (ROC) curve show the better prediction performance of the model compared to previous studies. The C-statistics of our model for OS prediction are 0.80, 0.83, and 0.81 in the training, validation, and overall cohorts, respectively, while for CSS prediction are 0.83, 0.85, and 0.84. The AUCs for 3- and 5-year OS are the same and are 0.81, 0.83, and 0.81 in the training, validation, and overall cohorts, respectively. The AUCs for 3- and 5-year CSS are 0.78 and 0.80, 0.79 and 0.80, and 0.79 and 0.80 in those three cohorts.

**Conclusions:** Our model shows the best prediction accuracy of the OS and CSS for patients with vulvar cancer (VC), which is of significant clinical practice value.

## Introduction

Primary vulvar cancer (VC) is a rare malignancy that accounts for about 5% of all gynecologic cancer cases, with more than 6,100 newly diagnosed cases yearly and a rising death rate trends in the United States, leading to more than 1,400 in 2020 to 1,500 in 2021 (1, 2). Furthermore, 90% of VC is squamous cell carcinoma (VSCC) (3).

The primary therapy for VSCC is surgical resection and radiotherapy with/without chemotherapy (3, 4). VC frequently spreads to the regional lymph nodes. The patients with regional lymph nodes involvement had worse survival (5). For VSCC with lymph node metastasis, lymphadenectomy and sentinel lymph nodes biopsy (SLNB) were both carried out. However, lymphadenectomy is associated with a high probability of complications (66–85%) that are the fundamental cause of death after surgery, such as wound breakdown, infection, lymphoceles, lymphedema, cellulitis, and erysipelas (6). After applying several new surgical techniques of lymphadenectomy in recent years, the morbidity after lymphadenectomy decreased in recent years but remains high (7). SLNB is less aggressive and has a lower complication occurrence rate and thus could prolong the survival of patients with VSCC, so it is preferred as the replacement of lymphadenectomy for well-selected patients with VSCC. Moreover, SLNB has a sensitivity of more than 95% to indicate lymph node involvement and a specialty of nearly 100% (8). So SLNB should be included as a predictor in nomograms for survival prediction. However, no nomograms for predicting the survival of the patients with VC have taken SLNB into account.

Nomograms for predicting the cancer-specific survival (CSS) of the patients with VC have been developed. For example, a nomogram based on age, American Joint Committee on Cancer (AJCC) T stage, invasion depth, margin status, and lymph nodes status had a Harrell's C concordance statistics (C-statistic) of 0.78 and 0.83 in the validation study and training study, respectively (9, 10). However, detection of margin status and the number of lymph nodes involved are difficult and highly influenced by the experience and imaging techniques of clinicians. Some studies even found that margin status was not associated with survival, probably due to the hardship of identifying margin status (11–13). A recently developed study comprising age, tumor size, pathological stage, metastasis, radiotherapy, chemotherapy, and surgery had a prediction accuracy of C-statistics of 0.81 for CSS prediction, without considering the invasion depth, SLNB, and N stage (14). Moreover, no nomograms have been developed to predict the overall survival (OS) of the patients with VC.

Therefore, we tried to develop novel nomograms for precisely predicting the OS and CSS for the patients with VC using a population-based database.

## Materials and Methods

### Data Source and Study Population

The patients with the International Classification of Diseases for Oncology, 3rd Edition (ICD-O-3) codes of C51.0, C51.1, C51.2, C51.8, C51.9, and the ICD-O-3 histology codes 8050-8084 (squamous cell carcinoma) were selected from the Surveillance, Epidemiology, and End Results (SEER) Program database of the National Cancer Institute from 2004 to 2016 (15). Moreover, the patients were excluded under the following conditions: (1) not squamous cell carcinoma; (2) not the only first primary tumor; (3) age at diagnosis <18 or more than 100 years old; and (4) not confirmed by positive histology.

The variables of primary site, year of diagnosis, insurance type, age, marital status, tumor size, pathological grade, radiotherapy, chemotherapy, historical stage, invasion depth, lymphadenectomy, sentinel lymph node biopsy, surgery, International Federation of Gynecology and Obstetrics (FIGO) stage, AJCC stage, AJCC T, N, and M stage were retrieved from the corresponding fields of the SEER database.

### Outcomes

Overall survival was the primary outcome. CSS was the secondary outcome, calculated based on the patients whose death was attributable to vulvar cancer, while those who died of other reasons rather than VC were considered censors.

### Statistical Analysis

The overall sample was randomly split into training (75%) and validation (25%) cohorts, with the constraints of keeping the proportion of death events similar between those two cohorts, following the Transparent Reporting of a multivariable prediction model for Individual Prognosis or Diagnosis (TRIPOD) guideline (16). The chi-square test was applied to test the balance of all the available variables between the training and validation groups. Within the training cohort, a 10-fold cross-validation with 200 repetitions was carried out to identify the best fit model based on the net benefit and Harrell's C-statistic, model with the largest average net benefit was considered as the best fit model. If the models have the same net benefit and C-statistic, the one with fewer variables is chosen.

After the best fit model had been identified, the model was refitted on the overall training cohort and validated on the validation cohort. The net benefit from the decision curve analysis (DCA), C-statistic of calibration plot, and areas under receiver operating characteristic curve (AUC) were used to measure the prediction performance of models. The 95% CI of C-statistic and AUC were calculated by bootstrap with 1,000 repetitions. The nomograms based on the best-fit model refitted on the overall sample (combination of training and validation cohort) were generated for 3- and 5-year OS and CSS.

Several multivariate Cox models such as all and part of the year of diagnosis, insurance status, age, race, marital status, primary site, historical stage, pathological grade, tumor size, invasion depth, surgery, radiotherapy, chemotherapy, FIGO stage, AJCC T, N and M stage were fitted and compared. Hazard ratio (*HR*) and their corresponding 95% CIs were calculated.

A two-tailed *p-*value of < 0.05 was considered statistically significant. All the statistics were performed in STATA 16.0 software (StataCorp, College Station, TX, USA).

## Results

### Baseline Characteristics

**Figure 1:**
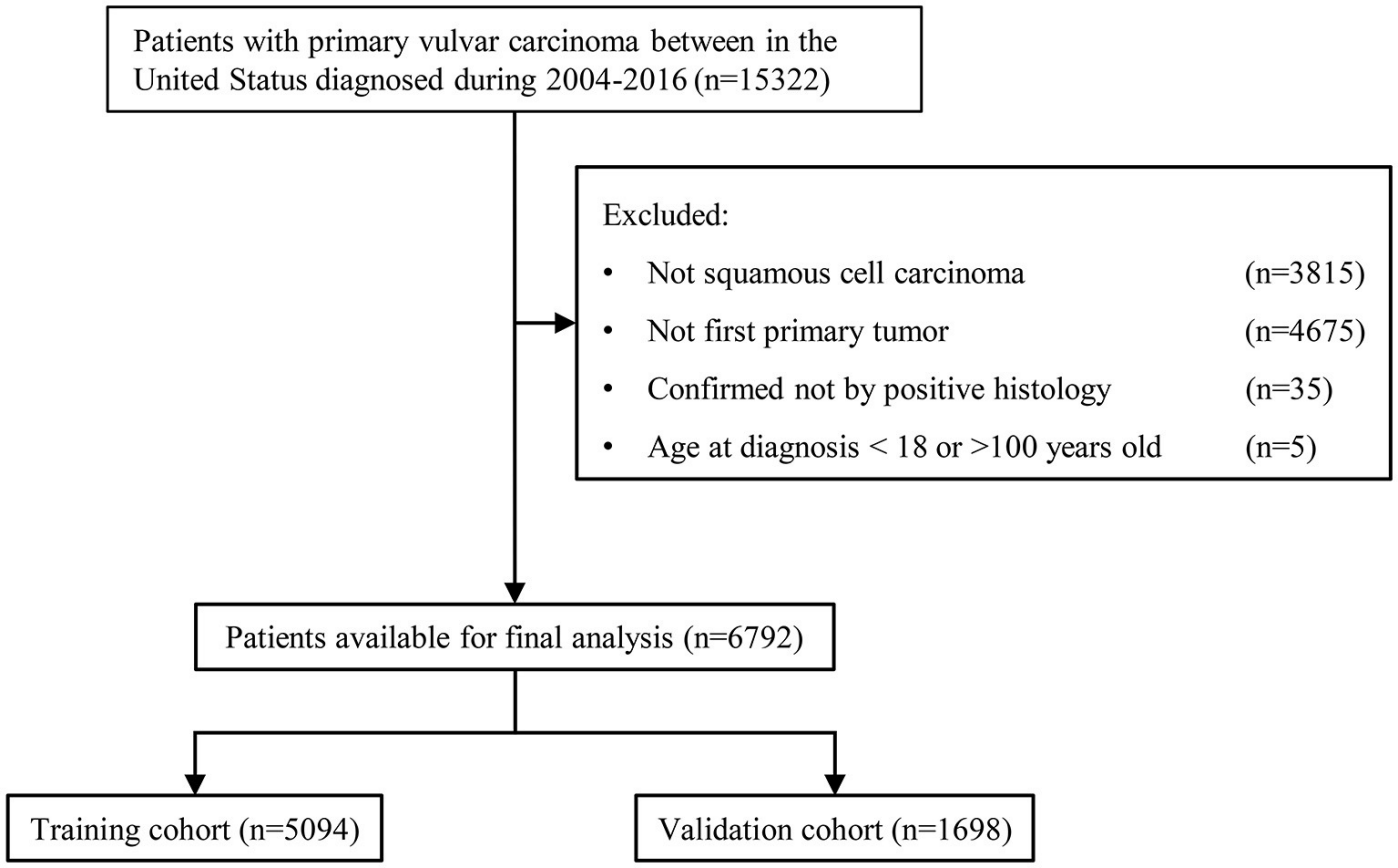
Flowchart of the patient selection procedure.

Figure 1 shows the sample selection procedure. Of the 6,792 patients in this study, 5,094 (75%) and 1,698 (25%) were randomly allocated into the training and validation groups. The primary site, year of diagnosis, insurance type, age, marital status, tumor size, pathological grade, radiotherapy, chemotherapy, historical stage, invasion depth, lymphadenectomy, sentinel lymph node biopsy, surgery, FIGO stage, AJCC stage, AJCC T, N and M stage were all balanced between the training and validation groups (all chi-square *p* > 0.05, Table 1).

**Table 1:** The baseline characteristics.

**Characteristics**	**Training cohort No. (%)**	**Validation cohort No. (%)**	***P*** **-value**
**Total**	5,094 (75)	1,698 (25)	
**Year of diagnosis**			0.638
2004–2009	2,084 (40.5)	659 (39.9)	
2010–2016	3,056 (59.5)	993 (60.1)	
**Primary site**			0.346
Labium majus	423 (8.2)	125 (7.6)	
Labium minus	238 (4.6)	68 (4.1)	
Clitoris	100 (1.9)	23 (1.4)	
Overlapping lesion of vulva	228 (4.4)	68 (4.1)	
Vulva, NOS	4,151 (80.8)	1,368 (82.8)	
**Age**			0.859
Median (IQR)	66 (53–79)	65 (53–79)	
18–39	258 (5.0)	85 (5.1)	
40–59	1,694 (33.0)	528 (32.0)	
60–79	1,949 (37.9)	643 (38.9)	
≥80	1,239 (24.1)	396 (24.0)	
**Insurance**			0.594
Insured	3,124 (60.8)	1,011 (61.2)	
Medicaid	675 (13.1)	225 (13.6)	
Uninsured	159 (3.1)	41 (2.5)	
Unknown	1,182 (23.0)	375 (22.7)	
**Marital status**			0.81
Married	1,856 (36.1)	598 (36.2)	
Single	932 (18.1)	297 (18.0)	
Divorced/separated/windowed	1,953 (38.0)	640 (38.7)	
Unknown	399 (7.8)	117 (7.1)	
**Tumor size**			0.999
<2 cm	1,464 (28.5)	471 (28.5)	
2–4 cm	1,239 (24.1)	399 (24.2)	
≥4 cm	1,375 (26.8)	443 (26.8)	
Unknown	1,062 (20.7)	339 (20.5)	
**Pathological grade**			0.176
Grade I	1,344 (26.1)	401 (24.3)	
Grade II	1,976 (38.4)	627 (38.0)	
Grade III/IV	836 (16.3)	271 (16.4)	
Unknown	984 (19.1)	353 (21.4)	
**Historic stage**			0.386
Localized	2,808 (54.6)	923 (55.9)	
Regional	1,879 (36.6)	569 (34.4)	
Distant	286 (5.6)	99 (6.0)	
Unstaged	167 (3.2)	61 (3.7)	
**Radiotherapy**			0.147
No	3,521 (68.5)	1,163 (70.4)	
Yes	1,619 (31.5)	489 (29.6)	
**Chemotherapy**			0.797
No/unknown	4,189 (81.5)	1,351 (81.8)	
Yes	951 (18.5)	301 (18.2)	
**Invasion depth**			0.846
<1 mm	584 (11.4)	190 (11.5)	
≥1 mm	1,733 (33.7)	568 (34.4)	
Unknown	2,823 (54.9)	894 (54.1)	
**Lymphadenectomy**			0.258
No	2,819 (54.8)	912 (55.2)	
Yes	2,279 (44.3)	733 (44.4)	
Unknown	42 (0.8)	7 (0.4)	
**SLNB**			0.125
No	4,755 (92.5)	1,520 (92.0)	
Yes	343 (6.7)	125 (7.6)	
Unknown	42 (0.8)	7 (0.4)	
**Surgery type**			0.146
None	1,036 (20.2)	331 (20.0)	
LTE	2,421 (47.1)	822 (49.8)	
Vulvectomy	667 (13.0)	185 (11.2)	
Debulking	1,016 (19.8)	314 (19.0)	
**FIGO stage**			0.809
I	1,838 (35.8)	599 (36.3)	
II	269 (5.2)	85 (5.1)	
III	772 (15.0)	230 (13.9)	
IV	1,008 (19.6)	320 (19.4)	
Unknown	1,253 (24.4)	418 (25.3)	
**AJCC stage**			0.926
I	2,129 (41.4)	691 (41.8)	
II	640 (12.5)	195 (11.8)	
III	892 (17.4)	276 (16.7)	
IVA	286 (5.6)	93 (5.6)	
IVB	195 (3.8)	69 (4.2)	
Unknown	998 (19.4)	328 (19.9)	
**T stage**			0.689
T1	2,970 (57.8)	963 (58.3)	
T2	1,134 (22.1)	348 (21.1)	
T3	471 (9.2)	151 (9.1)	
T4	71 (1.4)	18 (1.1)	
TX	494 (9.6)	172 (10.4)	
**N stage**			0.333
N0	3,616 (70.4)	1,165 (70.5)	
N1	711 (13.8)	227 (13.7)	
N2	510 (9.9)	150 (9.1)	
N3	54 (1.1)	27 (1.6)	
NX	249 (4.8)	83 (5.0)	
**M stage**			0.494
M0	4,828 (93.9)	1,543 (93.4)	
M1	209 (4.1)	78 (4.7)	
MX	103 (2.0)	31 (1.9)	
**Outcome**			0.500
Alive	3,099 (60.3)	1,002 (60.7)	
Dead attributable to vulvar cancer	1,290 (25.1)	396 (24.0)	
Dead of other causes	729 (14.2)	243 (14.7)	
Dead of unknown reason	22 (0.4)	11 (0.7)	
**Follow-up time (IQR), month**	20 (10–69)	30 (9–70)	

*IQR, interquartile range; SLNB, sentinel lymph node biopsy; FIGO, International Federation of Gynecology and Obstetrics; AJCC, American Joint Committee on Cancer*.

### Multivariate Cox Proportional Hazards Model Selection

Within the training cohort, a 10-fold cross-validation with 200 repetitions was carried out to choose the best fit model with the largest average net benefit. If two models have a similar average net benefit, the one with fewer variables was selected. Finally, the model comprising age, insurance, tumor size, pathological grade, radiotherapy, chemotherapy, invasion depth, lymphadenectomy, SLNB, surgery, N stage, and M stage was chosen for both predicting OS and CSS.

### Effects of Variables in the Best Fit Model for OS and CSS

As shown in Tables 2, 3, within the training cohort, age, insurance, tumor size, pathological grade, chemotherapy, invasion depth, SLNB, surgery, and N stage and M stage were the factors significantly associated with OS and CSS (all *p* < 0.001). However, radiotherapy was not associated.

**Table 2:** The results of multivariate cox proportional hazard risk model for overall survival (OS).

**Characteristics**	**Training cohort (*****N*** **=** **5,094)**		**Overall cohort (*****N*** **=** **6,792)**	
	**HR (95% CI)**	***p*** **-value**	**HR (95% CI)**	***p*** **-value**
**Age**				
18–39	Reference		Reference	
40–59	1.83 (1.25–2.67)	0.002	1.92 (1.38–2.67)	<0.001
60–79	4.35 (3.00–6.31)	<0.001	4.44 (3.20–6.17)	<0.001
≥80	9.59 (6.61–13.92)	<0.001	10.00 (7.20–13.88)	<0.001
**Insurance**				
Insured	Reference		Reference	
Medicaid	1.34 (1.16–1.55)	<0.001	1.32 (1.17–1.50)	<0.001
Uninsured	0.98 (0.69–1.40)	0.927	1.01 (0.74–1.36)	0.963
Unknown	1.27 (1.13–1.42)	<0.001	1.22 (1.10–1.34)	<0.001
**Tumor size**				
<2 cm	Reference		Reference	
2–4 cm	1.63 (1.42–1.88)	<0.001	1.65 (1.46–1.87)	<0.001
≥4 cm	1.96 (1.70–2.26)	<0.001	2.05 (1.81–2.32)	<0.001
Unknown	1.59 (1.36–1.87)	<0.001	1.60 (1.39–1.83)	<0.001
**Pathological grade**				
Grade I	Reference		Reference	
Grade II	1.13 (1.00–1.28)	0.047	1.12 (1.00–1.24)	0.044
Grade III/IV	1.32 (1.14–1.53)	<0.001	1.32 (1.16–1.50)	<0.001
Unknown	0.79 (0.68–0.93)	0.003	0.81 (0.70–0.92)	0.002
**Radiotherapy**				
No	Reference		Reference	
Yes	0.93 (0.81–1.07)	0.298	0.91 (0.81–1.03)	0.139
**Chemotherapy**				
No/unknown	Reference		Reference	
Yes	0.74 (0.63–0.87)	<0.001	0.71 (0.62–0.81)	<0.001
**Invasion depth**				
<1 mm	Reference		Reference	
≥1 mm	1.56 (1.28–1.91)	<0.001	1.57 (1.32–1.86)	<0.001
Unknown	1.65 (1.37–2.00)	<0.001	1.63 (1.38–1.92)	<0.001
**Lymphadenectomy**				
No	Reference		Reference	
Yes	0.71 (0.63–0.79)	<0.001	0.75 (0.68–0.84)	<0.001
Unknown	1.53 (0.93–2.52)	0.094	1.55 (1.00–2.42)	0.052
**SLNB**				
No	Reference		Reference	
Yes	0.60 (0.48–0.76)	<0.001	0.57 (0.46–0.70)	<0.001
**Surgery**				
None	Reference		Reference	
Yes	0.49 (0.42–0.57)	<0.001	0.45 (0.40–0.52)	<0.001
**N stage**				
N0	Reference		Reference	
N1	1.91 (1.65–2.21)	<0.001	1.94 (1.71–2.20)	<0.001
N2	2.69 (2.24–3.24)	<0.001	2.70 (2.31–3.16)	<0.001
N3	3.50 (2.50–4.89)	<0.001	3.06 (2.26–4.15)	<0.001
NX	1.36 (1.07–1.73)	0.014	1.40 (1.12–1.75)	0.004
**M stage**				
M0	Reference		Reference	
M1	2.04 (1.60–2.60)	<0.001	2.04 (1.67–2.49)	<0.001
MX	1.22 (0.88–1.70)	0.240	1.06 (0.77–1.47)	0.706

*HR, hazard ratio; SLNB, sentinel lymph node biopsy*.

**Table 3:** The results of multivariate cox proportional hazard risk model for cancer-specific survival (CSS).

**Characteristics**	**Training cohort (*****N*** **=** **5,094)**		**Overall cohort (*****N*** **=** **6,792)**	
	**HR (95% CI)**	***p*** **-value**	**HR (95% CI)**	***p*** **-value**
**Age**				
18–39	Reference		Reference	
40–59	1.73 (1.09–2.75)	0.021	1.65 (1.11–2.44)	0.012
60–79	3.80 (2.40–6.01)	<0.001	3.47 (2.35–5.10)	<0.001
≥80	7.36 (4.64–11.66)	<0.001	6.79 (4.61–10.02)	<0.001
**Insurance**				
Insured	Reference		Reference	
Medicaid	1.28 (1.08–1.52)	0.005	1.25 (1.07–1.45)	0.004
Uninsured	1.06 (0.70–1.60)	0.792	1.04 (0.73–1.49)	0.817
Unknown	1.27 (1.10–1.47)	0.001	1.21 (1.07–1.37)	0.002
**Tumor size**
<2 cm	Reference		Reference	
2–4 cm	1.86 (1.52–2.29)	<0.001	1.92 (1.60–2.30)	<0.001
≥4 cm	2.20 (1.79–2.71)	<0.001	2.33 (1.95–2.79)	<0.001
Unknown	1.89 (1.51–2.36)	<0.001	1.93 (1.59–2.34)	<0.001
**Pathological grade**				
Grade I	Reference		Reference	
Grade II	1.13 (0.97–1.33)	0.117	1.15 (1.00–1.32)	0.054
Grade III/IV	1.36 (1.13–1.64)	0.001	1.43 (1.22–1.68)	<0.001
Unknown	0.65 (0.53–0.80)	<0.001	0.70 (0.59–0.84)	<0.001
**Radiotherapy**				
No	Reference		Reference	
Yes	1.09 (0.91–1.29)	0.344	1.07 (0.92–1.24)	0.372
**Chemotherapy**				
No/unknown	Reference		Reference	
Yes	0.79 (0.66–0.94)	0.010	0.71 (0.61–0.83)	<0.001
**Invasion depth**				
<1 mm	Reference		Reference	
≥1 mm	1.72 (1.26–2.35)	0.001	1.77 (1.35–2.34)	<0.001
Unknown	1.77 (1.31–2.40)	<0.001	1.91 (1.46–2.50)	<0.001
**Lymphadenectomy**				
No	Reference		Reference	
Yes	0.84 (0.72–0.98)	0.027	0.90 (0.79–1.03)	0.126
Unknown	1.56 (0.79–3.10)	0.201	1.58 (0.92–2.72)	0.094
**SLNB**				
No	Reference		Reference	
Yes	0.54 (0.39–0.75)	<0.001	0.55 (0.42–0.73)	<0.001
**Surgery**				
None	Reference		Reference	
Yes	0.39 (0.32–0.47)	<0.001	0.38 (0.32–0.45)	<0.001
**N stage**				
N0	Reference		Reference	
N1	2.36 (1.98–2.81)	<0.001	2.43 (2.09–2.82)	<0.001
N2	3.38 (2.77–4.14)	<0.001	3.47 (2.93–4.12)	<0.001
N3	3.99 (2.70–5.90)	<0.001	3.80 (2.72–5.32)	<0.001
NX	1.72 (1.27–2.33)	<0.001	1.85 (1.42–2.43)	<0.001
**M stage**				
M0	Reference		Reference	
M1	2.18 (1.69–2.80)	<0.001	2.28 (1.85–2.81)	<0.001
MX	1.00 (0.66–1.50)	0.984	0.91 (0.62–1.33)	0.618

*HR, hazard ratio; SLNB, sentinel lymph node biopsy*.

### Model Prediction Accuracy

**Figure 2:**
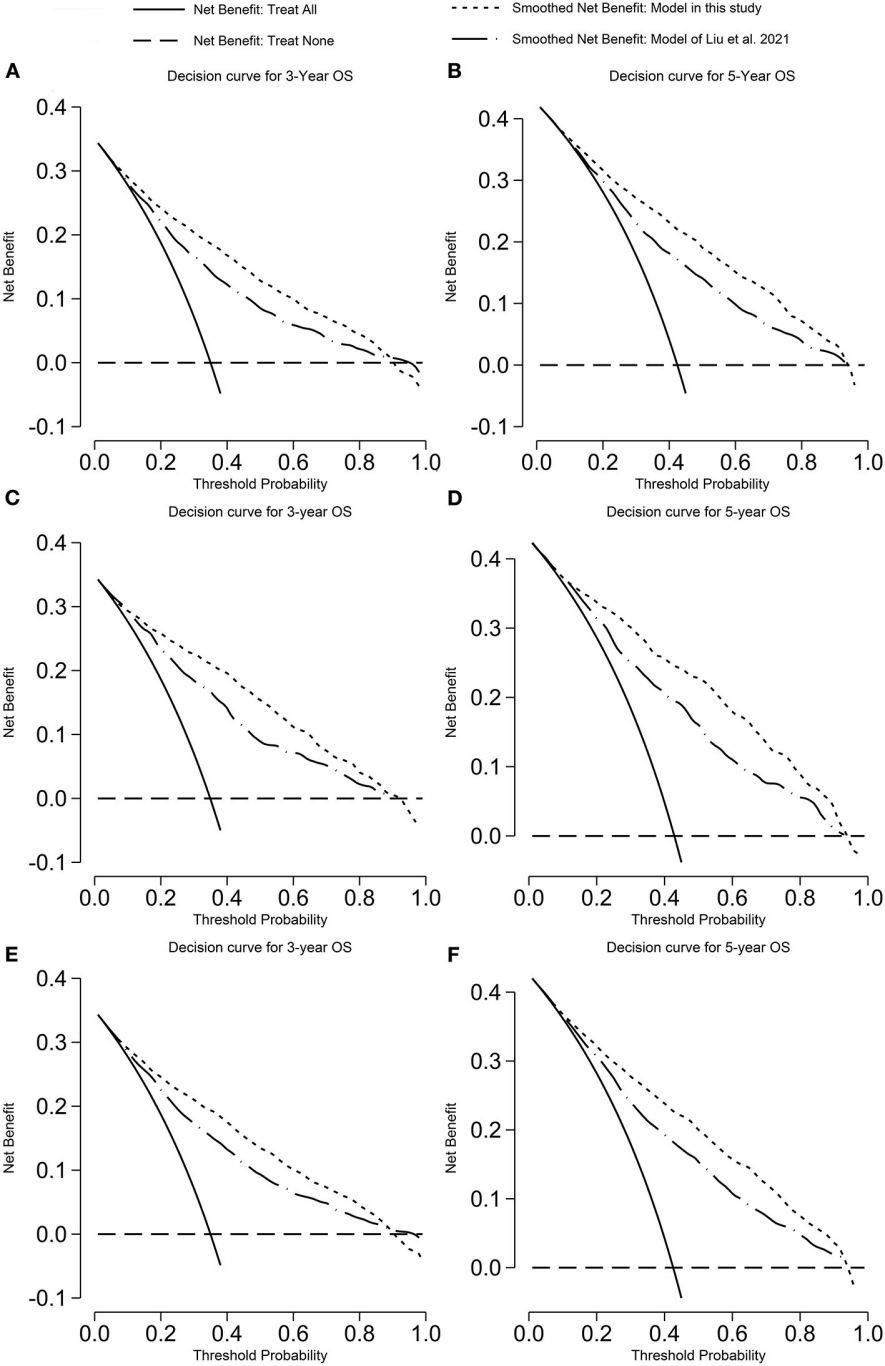
Decision curve analysis on the model predicting OS. **(A)** 3-years OS in the training cohort; **(B)** 5-years OS in the training cohort; **(C)** 3-years OS in the validation cohort; **(D)** 5-years OS in the validation cohort; **(E)** 3-years OS in the overall cohort; and **(F)** 5-years OS in the overall cohort. OS, overall survival.

**Figure 3:**
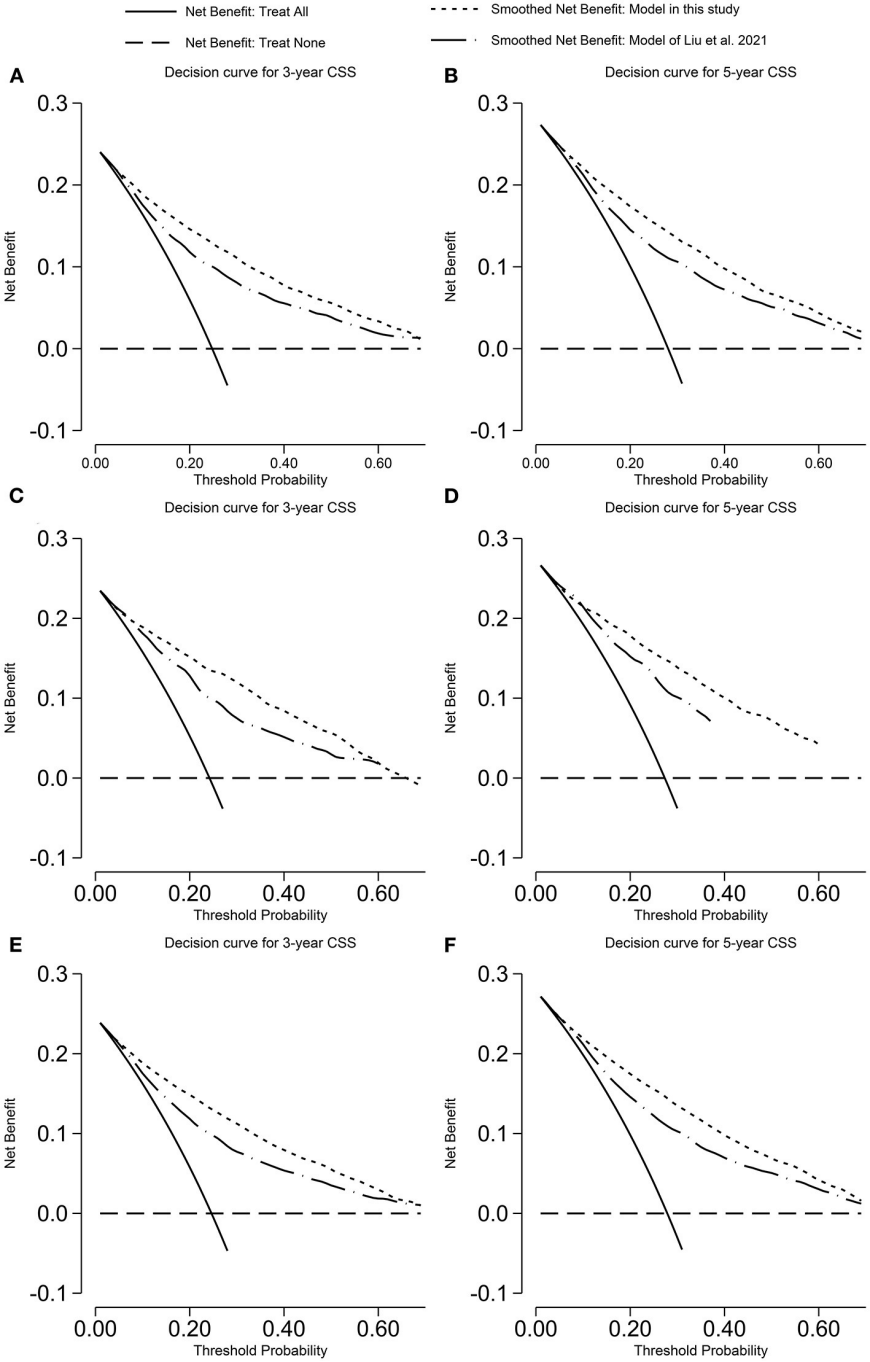
Decision curve analysis on the model predicting CSS. **(A)** 3-years CSS in the training cohort; **(B)** 5-years CSS in the training cohort; **(C)** 3-years CSS in the validation cohort; **(D)** 5-years CSS in the validation cohort; **(E)** 3-years CSS in the overall cohort; and **(F)** 5-years CSS in the overall cohort. CSS, cancer-specific survival.

The DCA plot was shown in Figures 2, 3. Compared with the previously published nomograms with the best accuracy for CSS prediction (14), our model has the larger net benefit.

**Figure 4:**
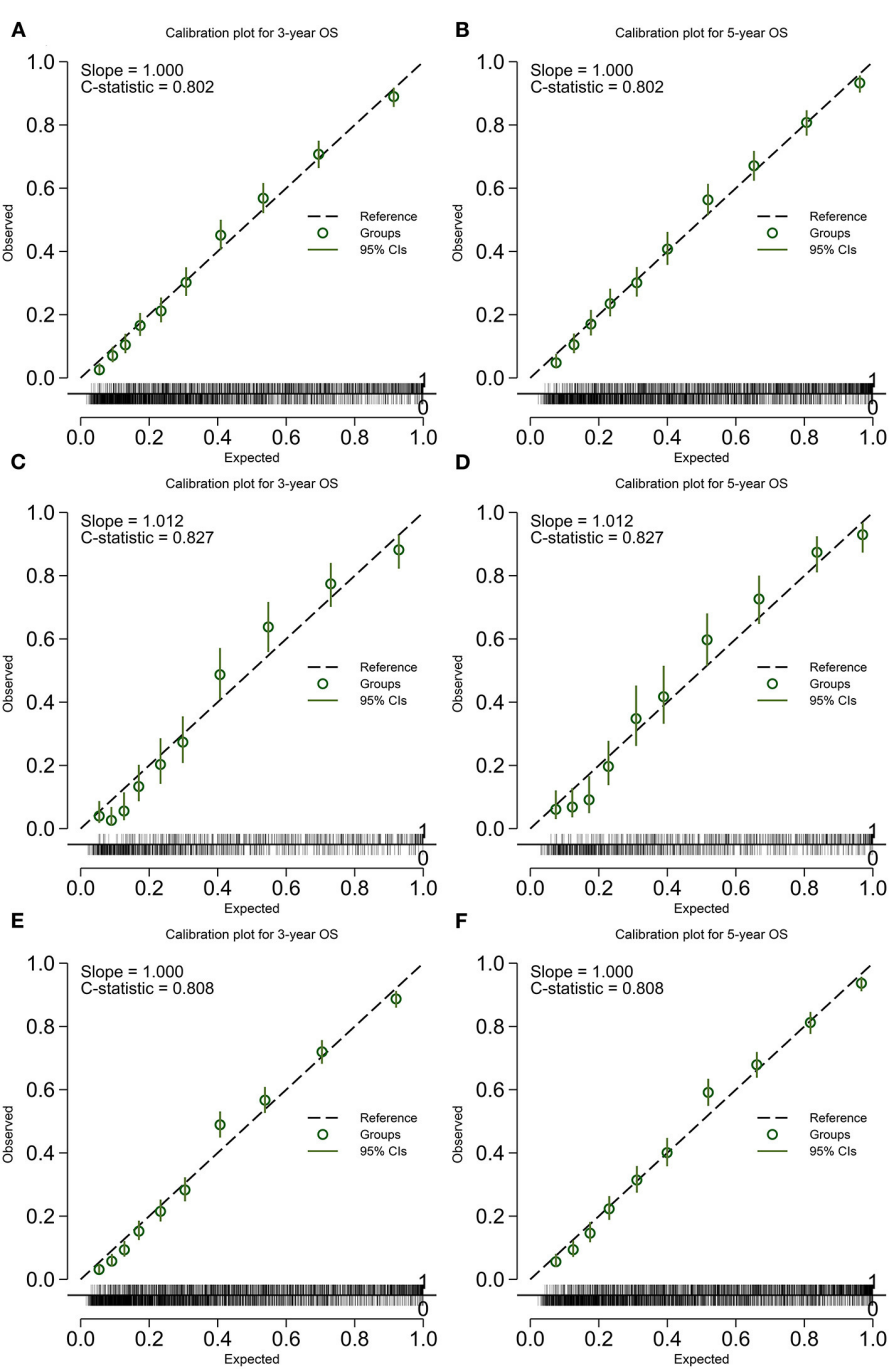
Calibration plot of the model predicting OS. **(A)** 3-years OS in the training cohort; **(B)** 5-years OS in the training cohort; **(C)** 3-years OS in the validation cohort; **(D)** 5-years OS in the validation cohort; **(E)** 3-years OS in the overall cohort; and **(F)** 5-years OS in the overall cohort. OS, overall survival.

**Figure 5:**
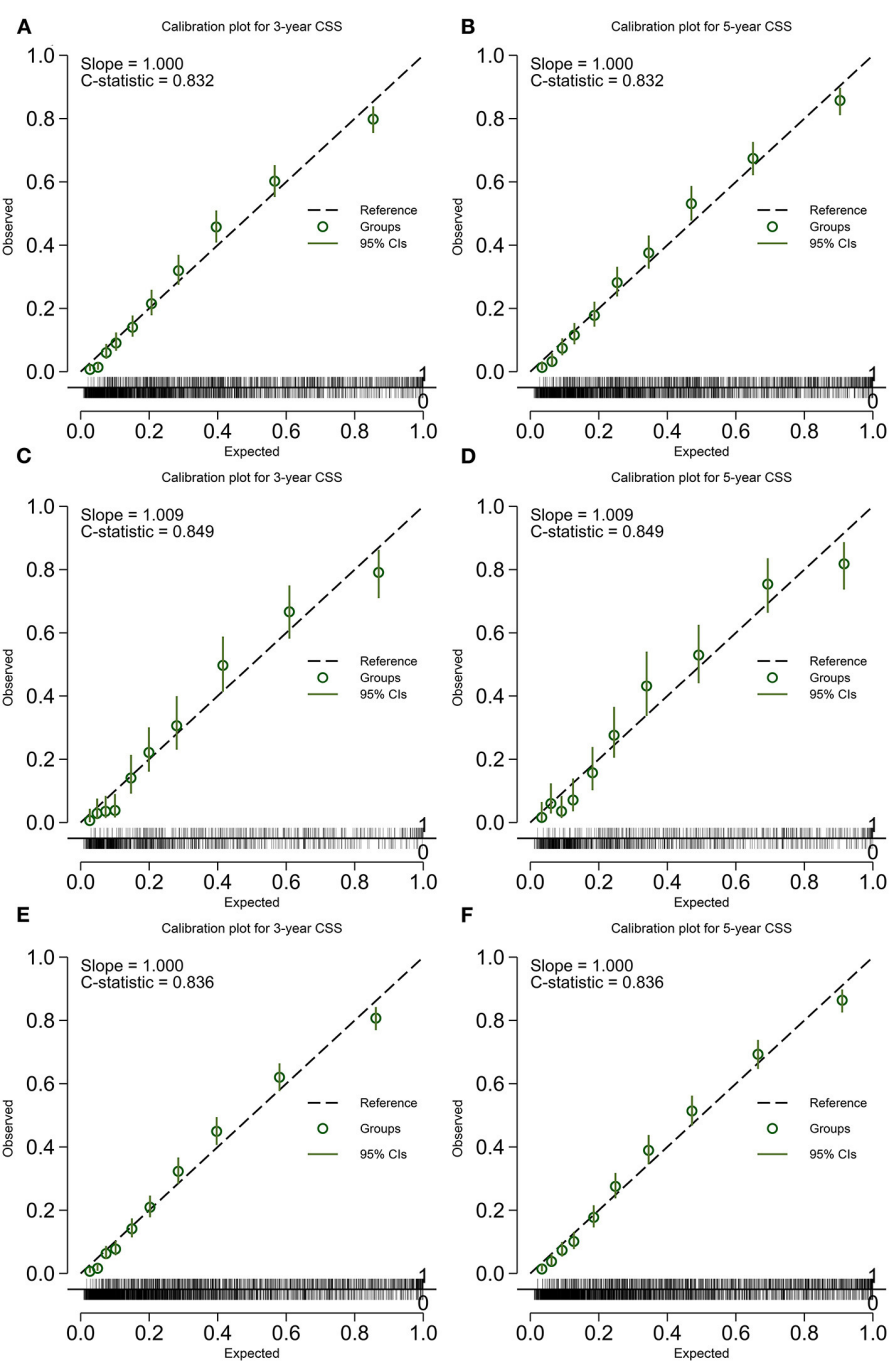
Calibration plot of the model predicting CSS. **(A)** 3-years CSS in the training cohort; **(B)** 5-years CSS in the training cohort; **(C)** 3-years CSS in the validation cohort; **(D)** 5-years CSS in the validation cohort; **(E)** 3-years CSS in the overall cohort; and **(F)** 5-years CSS in the overall cohort. CSS, cancer-specific survival.

The calibration plot was displayed in Figures 4, 5. The model has C-statistics of 0.80 (95% CI 0.79–0.81), 0.83 (95% CI 0.81–0.84), and 0.81 (95% CI 0.80–0.82) for the training cohort, validation cohort, and overall cohort, respectively, for predicting the OS. For predicting the CSS, those numbers are 0.83 (95% CI 0.82–0.84), 0.85 (95% CI 0.83–0.86), and 0.84 (95% CI 0.83–0.84).

**Figure 6:**
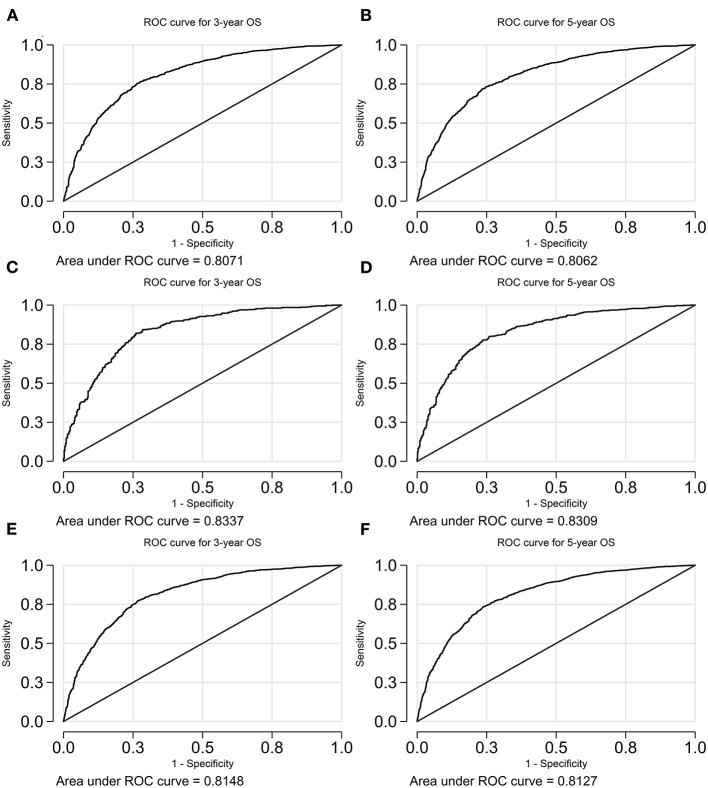
A receiver operating characteristic (ROC) curve of the model predicting OS. **(A)** 3-years OS in the training cohort; **(B)** 5-years OS in the training cohort; **(C)** 3-years OS in the validation cohort; **(D)** 5-years OS in the validation cohort; **(E)** 3-years OS in the overall cohort; and **(F)** 5-years OS in the overall cohort. OS, overall survival.

**Figure 7:**
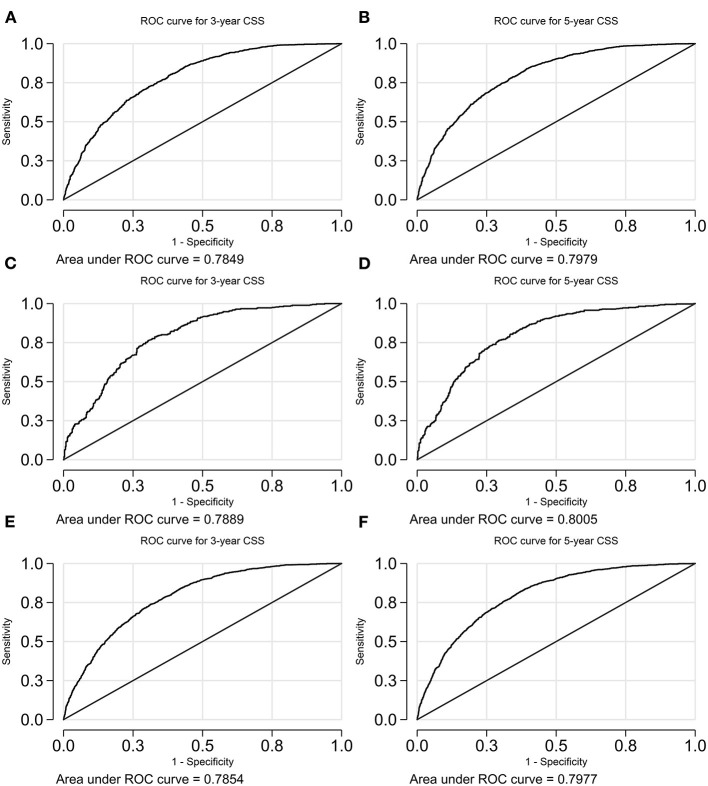
A ROC curve of the model predicting CSS. **(A)** 3-years CSS in the training cohort; **(B)** 5-years CSS in the training cohort; **(C)** 3-years CSS in the validation cohort; **(D)** 5-years CSS in the validation cohort; **(E)** 3-years CSS in the overall cohort; and **(F)** 5-years CSS in the overall cohort. CSS, cancer-specific survival.

The receiver operating characteristic (ROC) curves were illustrated in Figures 6, 7. The AUCs for the prediction of 3- and 5-year OS are 0.81 (95% CI 0.79–0.82) and 0.81 (95% CI 0.79–0.82), 0.83 (95% CI 0.81–0.86) and 0.83 (95% CI 0.81–0.85), and 0.81 (95% CI 0.80–0.83) and 0.81 (95% CI 0.80–0.82), respectively, for the training cohort, validation cohort, and the overall cohort. With reference to the prediction of CSS, those numbers are 0.78 (95% CI 0.77–0.80) and 0.80 (95% CI 0.78–0.81), 0.79 (95% CI 0.76–0.82) and 0.8 (95% CI 0.77–0.83), and 0.79 (95% CI 0.77–0.80) and 0.80 (95% CI 0.79–0.81), respectively.

### Nomogram for Predicting 3- and 5-Year Survival

**Figure 8:**
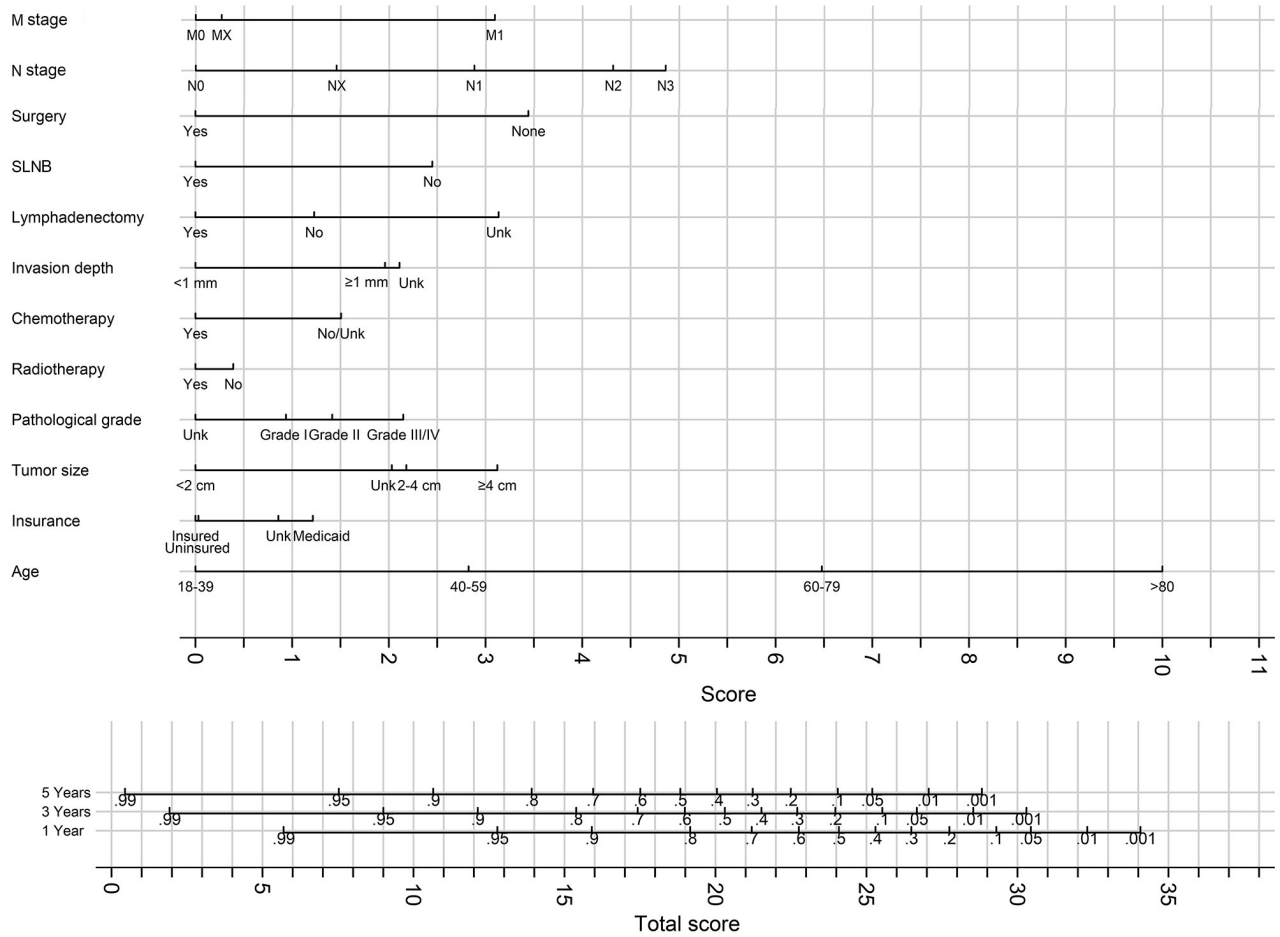
The nomograms for predicting 3- and 5-years OS. Unk, unknown.

**Figure 9:**
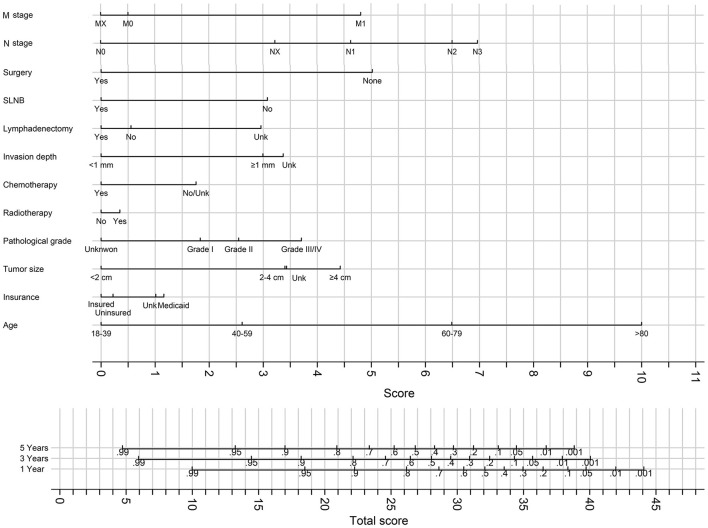
The nomograms for predicting 3- and 5-years CSS. Unk, unknown.

The best fit model was refitted on the overall cohort (combination of the training and validation cohort), and the result of the model was used to generate nomograms for predicting 3- and 5-year OS (Figure 8) and CSS (Figure 9).

## Discussion

This study developed novel nomograms to predict the 3- and 5-year OS and CSS for the patients with VSCC aged 18–100 years, based on a cohort of 6,792 cases from a population-based multicenter database. To our knowledge, the novel nomograms in our study have the best prediction accuracy, with excellent clinical practice importance.

Compared with the previously developed nomograms for predicting CSS of the patients with VC, for CSS prediction, our model has the better net benefit and the largest C-statistics of 0.83, 0.85, and 0.84 in the training, validation, and overall cohort, respectively. Our model comprises of factors that are commonly inspected and easy to get in clinical practice. Moreover, we did not exclude cases with the variables having missed or unknown values, which expanded the applying range of our model.

In line with previous studies, our model comprises age, tumor size, pathologic grade, radiotherapy, chemotherapy, surgery, and M stage, which were significant factors associated with CSS and included in the previously generated nomograms (9, 10, 14). Moreover, the FIGO stage was not included in our final model, although it was the most prevalent stage system for gynecological cancers, similar to two studies (9, 10). The invasion depth and N stage were also included in the final model, which has been argued as an essential prognostic factor of VSCC (9, 10) but not included in a more recently published nomogram (14). The inclusion of radiotherapy tended to be associated with improved survival; although it is not significant in the final model, the addition of it increased the prediction accuracy of the model, contrary to a recent study in which radiotherapy was negatively associated with VSCC (14).

To our knowledge, the first unique characteristic of this study is that it is the first study that generated a nomogram for predicting 3- and 5-year OS of the patients with VSCC. The nomogram for OS prediction had a good prediction accuracy measured by C-statistics of 0.80, 0.83, and 0.81 in the training, validation, and overall cohorts, respectively. In our study, the models for predicting OS and CSS include the same variables. Accordingly, once the variables of predicting CSS have been determined, OS can be predicted, which intensifies the application of our nomograms. The second unique characteristic is that we included SLNB and lymphadenectomy in the novel nomograms, and those two variables were statistically significant in the best fit model, which led to the precise prediction of the survival and adaption to modern surgical technique development. SLNB and lymphadenectomy have never been integrated as a predictor in nomograms for predicting the survival of patients with VSCC. However, the beneficial role of SLNB in improving survival has been proved in previous studies (17–19). The inclusion of SLNB in the model has improved the prediction accuracy considerably. Age and N stage was the strongest predictors of OS and CSS in our model, followed by surgery, M stage, and tumor size.

Before applying those nomograms in clinical practice, several points need to clarify. First, we only included VC patients with squamous cell carcinoma in the training and validation procedures, and accordingly, those nomograms could only be used for patients with VSCC. Applying to other histological types of patients with VC is not suggested. Second, the models were built based on the patients with VC aged between 18 and 100 years old. Whether those can be expanded to patients older than 100 has not been straightforward; thus, expansion should be cautious. Third, the VC patients with other malignancies or not with VSCC as a first tumor were not included in the training and validation samples; accordingly, the novel nomograms should not be applied to those patients. In addition, the nomograms in this study should be preferred to be applied to the patients with just one malignancy of VSCC. Fourth, the patients with VSCC confirmed not by positive histology were not suitable for those nomograms due to the exclusion of those patients from the sample.

This study has some limitations. First, we could not obtain detailed information on radiotherapy and chemotherapy, for example, the drug agent and dose of chemotherapy and the intensity of radiotherapy. Thus, we could not control the impact of those factors on survival. Second, due to the nature of a retrospective study, there might be missing essential factors for predicting survival, which would lead to bias. Third, the usefulness of those nomograms may be limited to the United States because we used the SEER database, which only includes the United States population. Fourth, we could not carry out external validation because no patients with VSCC from a different population or within a single center could be available as a result of the extreme rareness of VC.

Though our study had some limitations, it generated the nomograms with the best prediction accuracy and, first, predicting the OS of the patients with VSCC. This study provides the novel nomograms for the clinicians to accurately predict the OS and CSS of the patients with VSCC and, consequently, clinicians could carry out more targeted therapy procedures.

## Conclusions

The novel nomograms for predicting OS and CSS of the patients with VC have the best prediction accuracy, which is of significant clinical practice value.

## Data Availability Statement

Publicly available datasets were analyzed in this study. This data can be found here: https://seer.cancer.gov/.

## Ethics Statement

Ethical review and approval was not required for the study on human participants in accordance with the local legislation and institutional requirements. Written informed consent for participation was not required for this study in accordance with the national legislation and the institutional requirements.

## Author Contributions

WZ: conceptualization, methodology, data curation, formal analyses, supervision, writing original draft preparation, writing, reviewing, and editing. YY: formal analyses, methodology, software, supervision, visualization, writing original draft preparation, writing, reviewing, and editing. All authors contributed to the article and approved the submitted version.

## Conflict of Interest

The authors declare that the research was conducted in the absence of any commercial or financial relationships that could be construed as a potential conflict of interest.

## Publisher's Note

All claims expressed in this article are solely those of the authors and do not necessarily represent those of their affiliated organizations, or those of the publisher, the editors and the reviewers. Any product that may be evaluated in this article, or claim that may be made by its manufacturer, is not guaranteed or endorsed by the publisher.
